# Spectra without stories: reporting 94% dark and unidentified ancient proteomes

**DOI:** 10.12688/openreseurope.17225.1

**Published:** 2024-04-15

**Authors:** Yun Chiang, Frido Welker, Matthew James Collins

**Affiliations:** 1 Globe Institute, University of Copenhagen, Copenhagen, Denmark; 2 The Nice Institute of Chemistry, Universite Cote d'Azur, Nice, France; 3 McDonald Institute for Archaeological Research, University of Cambridge, Cambridge, England, UK

**Keywords:** palaeoproteomics, DDA, shotgun proteomics, database searching, bioinformatics challenges

## Abstract

**Background:**

Data-dependent, bottom-up proteomics is widely used for identifying proteins and peptides. However, one key challenge is that 70% of fragment ion spectra consistently fail to be assigned by conventional database searching. This ‘dark matter’ of bottom-up proteomics seems to affect fields where non-model organisms, low-abundance proteins, non-tryptic peptides, and complex modifications may be present. While palaeoproteomics may appear as a niche field, understanding and reporting unidentified ancient spectra require collaborative innovation in bioinformatics strategies. This may advance the analysis of complex datasets.

**Methods:**

14.97 million high-impact ancient spectra published in Nature and Science portfolios were mined from public repositories. Identification rates, defined as the proportion of assigned fragment ion spectra, were collected as part of deposited database search outputs or parsed using open-source python packages.

**Results and Conclusions:**

We report that typically 94% of the published ancient spectra remain unidentified. This phenomenon may be caused by multiple factors, notably the limitations of database searching and the selection of user-defined reference data with advanced modification patterns. These ‘spectra without stories’ highlight the need for widespread data sharing to facilitate methodological development and minimise the loss of often irreplaceable ancient materials. Testing and validating alternative search strategies, such as open searching and de novo sequencing, may also improve overall identification rates. Hence, lessons learnt in palaeoproteomics may benefit other fields grappling with challenging data.

## Introduction

Tandem mass spectrometry (LC-MS/MS) has been widely used for untargeted or shotgun proteomics. A standard method for automated fragment ion spectra (MS2) generation is data-dependent acquisition (DDA). In a typical DDA experiment, precursor ions are selected for fragmentation using their intensities observed in full MS1 scans and user-defined parameters (the top *N* method) ^1^. Three common fragmentation techniques include electron-transfer dissociation (ETD), collision-induced dissociation (CID), and higher-energy collisional dissociation (HCD); the latter two methods typically generate b- and y- ions due to the breakages of amide bonds during collision ^2^. The resulting MS2 data are clean and ready for peptide-spectrum matching and database searching, as fragments are generally derived from a single precursor within a small isolation window below 2 mass-to-charge ( *m/z*) ratios ^3^. Overall, data-driven shotgun proteomics has been robust and provides high–resolution data.

The combination of DDA and database searching has been a powerful tool for the deep and global discoveries of proteomes. However, DDA is susceptible to the co-fragmentation of co-eluting, near-isobaric precursor ions, which may generate complex chimeric spectra ^4^. Moreover, due to the stochastic nature of precursor sampling in DDA, peptide-level repeatability in technical replicates is reported to be below 60%, with low-abundance precursors often being under-sampled ^5^. While features like dynamic exclusion (a mass spectrometer parameter that temporarily excludes most-abundant precursors), and match-between-runs (MBR, a bioinformatics algorithm that realigns unidentified peaks using retention time and other MS1 features) have been designed to mitigate the missing value problem ^6, 7^, it has been estimated that only 30% of DDA MS2 spectra may be identified using database searching ^8^. This value is corroborated by the experimental observation of two large-scale analyses that just over 25% of MS2 spectra submitted to PRIDE and MassIVE are identified ^9, 10^.

Additionally, the efficiency of database searching may exacerbate the problem of unassigned spectra, leading to even lower rates of successful identification. This is primarily due to the limitations of the search space of a peptide matching algorithm, which is determined by a user-provided protein sequence database, digestion patterns, missed cleavages, a range of allowed peptide length, a predefined set of post-translational modifications (PTMs), and the mass tolerances of both precursors and fragment ions ^11^. Since acquired MS2 data are searched against theoretical spectra generated *in silico*, sequences not covered by a database, miscleaved peptides, and large mass shifts due to unexpected PTMs will be overlooked during conventional database searching. The search space also affects the filtering of peptide-spectrum matches (PSMs), as target-decoy competition is widely used to calculate false discovery rates (FDRs), with decoys generated based on the same parameters as the target database ^12^.

These limitations are pronounced in fields where managing the optimal search space is challenging due to the lack of *a priori* knowledge, non-model organisms, non-canonical variants, and the heterogeneity of PTMs. For example, in metaproteomics, it is difficult to anticipate which species may be present in samples, and not all microbial sequences are fully documented ^13^. The search space is also substantial in immunopeptidomics due to variants and the non-tryptic nature of human leukocyte antigen (HLA) molecules, particularly class I peptides ^14, 15^. As a result, the average identification rates of HLA data are around 10%. Similarly, it has been highlighted that less than 5 % of the phosphoproteome is identified, since phosphorylation sites are complex and vary in abundance ^16^.

These data analysis challenges are often summarised as the “dark matter of shotgun proteomics” ^9, 10, 17^. While similar issues are anticipated in palaeoproteomics, given that ancient proteins may be fragmented, non-tryptic, heavily modified, low in abundance, and high in non-model organisms that are not covered by standard reference databases, it remains unclear how many ancient spectra are dark and unassigned. This research question is significant for three reasons. Firstly, the study of ancient proteins has provided invaluable information for constructing narratives about diets, culinary practices, social interactions, and evolutionary histories in the *longue durée*. Yet, these unassigned ancient mass spectrometry data are, figuratively, spectra without stories. They are untapped reservoirs of amino acid substitutions, novel peptides, and complex PTMs that almost certainly conceal insights into the human past and protein preservation pathways.

Secondly, archaeological materials are unique and often irreplaceable artefacts. Since ancient proteins need to be extracted, destructive sampling is inevitable without the development and optimisation of non-destructive extraction protocols. It is crucial to evaluate the efficiency of palaeoproteomics analysis considering the associated trade-offs between potential scientific gains and irreversible loss of valuable ancient materials. Lastly, the challenges of working with complex, damaged, and low-abundance ancient proteomes may require innovation in data acquisition, database management, scoring algorithms, and search strategies. These advancements may increase current analytical capabilities and may be applied to other fields dealing with similarly challenging datasets that require a large search space.

## Methods

To address the question, we mined 15 high-impact datasets recently reviewed ^18^ and published in *Science* and *Nature Portfolio* journals ^19– 33^, resulting in the collection of 14.97 million deposited, publicly available ancient MS2 spectra. These datasets represent the best practice of the field and the challenges of analysing a diverse range of unique ancient materials, including bones, teeth, dental calculus, paintings, and ceramic pots. Additional criteria were that only complete datasets that cover original peak lists (.mgf files) and search results (summary.txt or .mzid files) were included.

Specifically, Orbitrap™ hybrid mass analysers (Q Exactive™ or Exploris™ instruments), DDA, and HCD were employed by all 15 datasets. 73.33% (11/15) of the search results were obtained from MaxQuant ^34^ while the remaining datasets (4/15) were analysed by Mascot ^35^. Overall identification rates, defined as the ratios of total assigned MS2 spectra to submitted MS2 spectra, were collected as part of submitted MaxQuant outputs. For Mascot results, open-source Pyteomics ^36^ was used to parse deposited .mzid outputs and determine the ratios of identified spectra. To contrast these identification rates of ancient proteome datasets with the general trend of identified MS2 spectra in public repositories, we obtained this metric from two recent meta-clustering analyses; these are the average identification rate of the PRIDE MS2 data (n= 256 million) ^9^ and that of the MassIVE depository (n= 669 million) ^10^. All figures were produced by non- proprietary python packages (Matplotlib ^37^/Seaborn ^38^) and annotated using Affinity Designer 2 ^39^. A free alternative for annotation is Inkscape.

## Results and discussion

**Figure 1.**
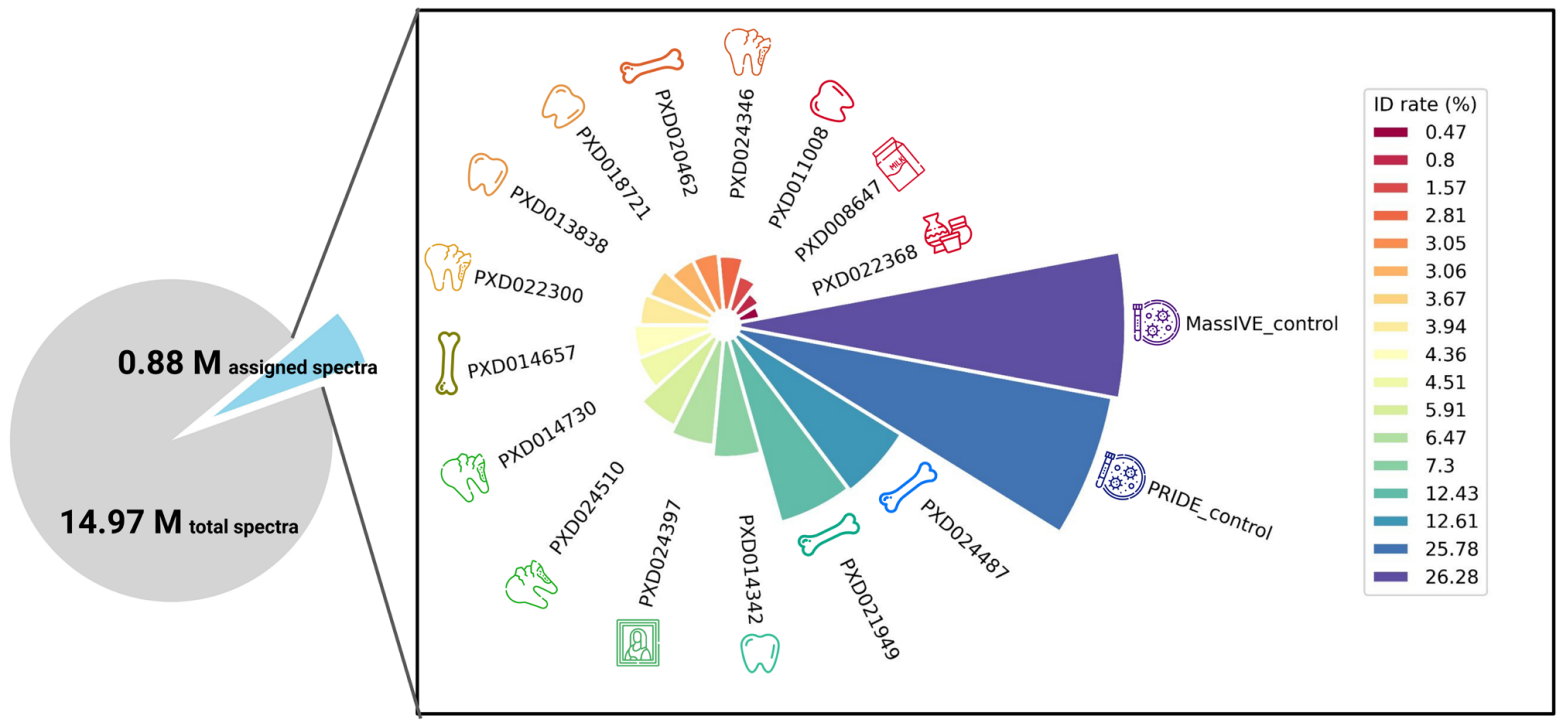
A pie chart of the total collected (14.97 million) and assigned MS2 spectra (0.88 million). Across all the 15 palaeoproteomics datasets containing skeletal, painting, and pottery samples, the overall identification rates (ID rate %) are low in comparison to two large-scale proteomics analyses (over 25%).

We report that only 0.88 out of 14.97 million total submitted MS2 spectra are identified, indicating that 94.12% of the queries remain uncharacterised. The identification rates of palaeoproteomics datasets range from 0.47% to 12.61% ( Figure 1). All of them consistently fall below the average identification rate of MS2 datasets deposited in PRIDE (25.78%) and MassIVE (26.28%). It should be noted that these identified MS2 spectra in the ancient datasets include peptides from both putative ancient proteins and contaminants, such as trypsin and human skin keratins. The validation of ancient proteins and how to effectively separate them from modern contaminants are ongoing debates ^27^. However, it is assumed that the actual proportion of genuinely assigned ancient spectra is below the average identification rate of 5.88%.

**Figure 2.**
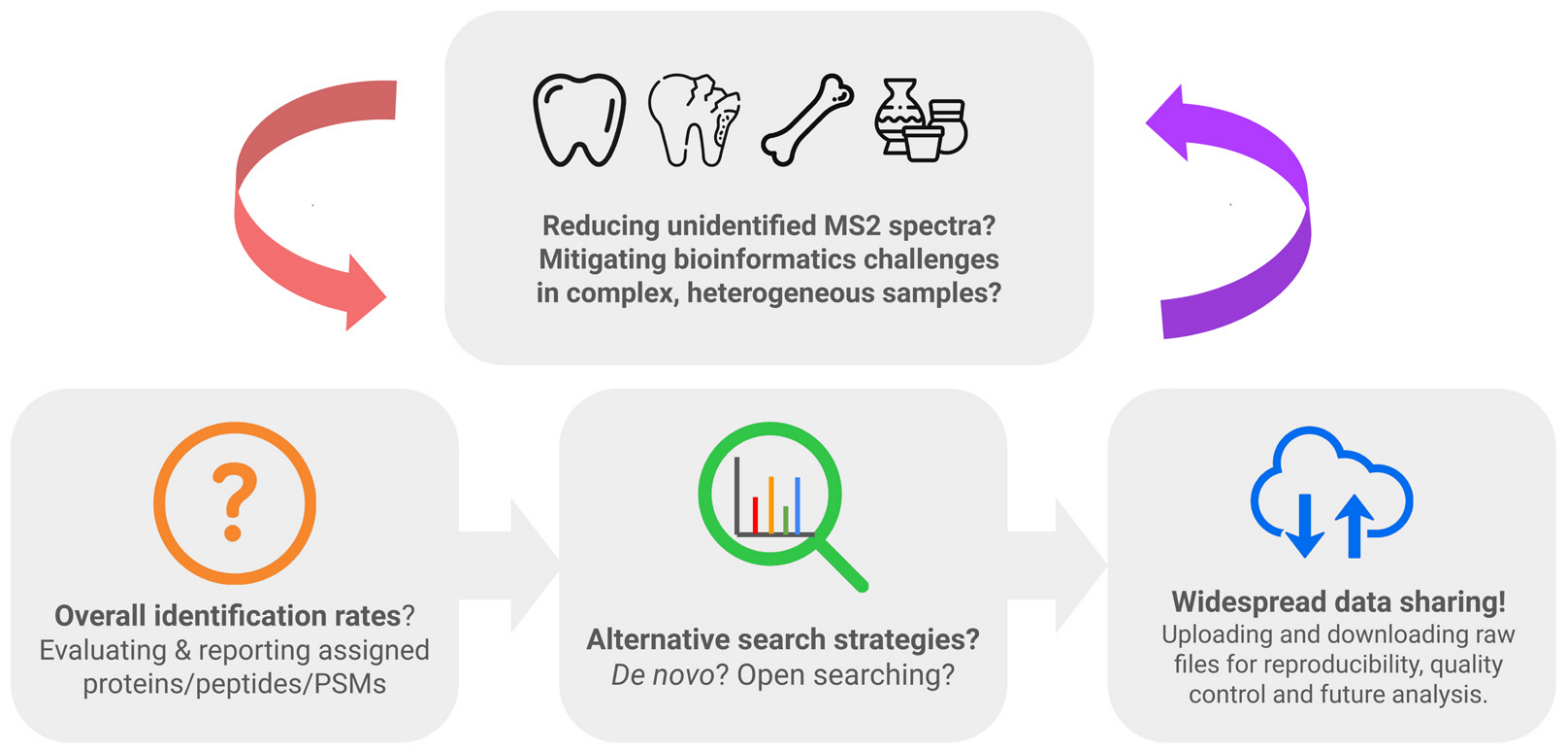
A flowchart that summarises key bottlenecks and suggested best practices to deal with the proteomics analysis of complex and heterogeneous samples.

Given that only 6% of the ancient MS2 spectra were successfully assigned, the 94% of dark, unidentified ancient proteomes highlights the need for widespread data sharing to facilitate methodological development and the re-evaluation of bioinformatics challenges inherent in the analyses of archaeological, palaeoanthropological, and palaeontological materials. Sharing data enables the re-analysis of often irreplaceable ancient materials, and fosters collaborative efforts in addressing the analytical challenges of complex archaeological data as seen in other related fields, especially ancient DNA ^40, 41^. While we acknowledge that the analysis of ancient MS2 data is often context-specific, depending on protein chemistry, preservation pathways, research questions, and the design of search space, we call for ongoing efforts to build comprehensive databases, refine search strategies for PTMs and ensure widespread data dissemination, to optimise search strategies and increase overall identification rates (summarised in Figure 2).

### How to build a relevant database?

Indeed, creating a comprehensive database for ancient proteins is significantly challenging without *a priori* information. Generally, ancient proteins may be derived from proteinaceous tissues, microbial communities, and past human activities, including cooking, crafting, parchment making, and leather tanning. An ideal database for searching includes all potential candidates without additional protein sequences. However, given the diversity and complexity of ancient proteins, often an entire UniProt knowledge database (Swiss-Prot and TrEMBL), up to about 251 million proteins and/or curated databases, typically consisting of hundreds of protein targets, are used in the database searching of ancient MS2 data ^18^.

Using an extensive database like the entire UniProt generates a substantial pool of candidates and increases the search space exponentially. The issues with a large search space are well documented in metaproteomics communities where NCBI RefSeq (over 113 million non-redundant proteins) have been used for the annotation of complicated protein systems ^42^. It has been reported that the utilisation of large databases (over 1 million sequences) results in decreased PSMs, increased risks of false positives, and difficulties in using FDRs to control for confident identification ^43^. In contrast, a focused and targeted approach may inadvertently raise false positive rates and may not capture the full spectrum of ancient proteins present in samples. Hence, a paradox is prevalent in palaeoproteomics: the need to balance the comprehensiveness of a database to maximise identification against the risk of increasing false positives and computational complexity.

A multi-step approach to database management may be beneficial; in metaproteomics, this generally includes an initial survey of a broad database to identify targets, followed by a subsequent run using a narrowed-down database ^44^. Machine learning and *de novo* sequencing without databases are other alternatives. Since novel and extinct sequences may be present in ancient samples, a *de novo*-centric approach may discover amino acid substitutions relevant to evolutionary studies and improve overall proteome composition ^45, 46^. Validation and optimisation may be required for these alternative approaches, and a substantial collection of quality ancient spectra is needed to train relevant machine learning models. Despite these hurdles, such alternative strategies may mitigate the limitations of database management.

### How to manage protein degradation & PTMs?

Apart from selecting a suitable database, the preservation states of ancient proteins may enlarge search space due to the increasing number of allowed missed cleavages, semi-tryptic/non-specific searching, and a wide range of associated PTMs. While in living organisms, PTMs mediate protein functions, structures, and interactions ^47^, in the context of palaeoproteomics, PTMs provide insights on the incorporation of proteins into archaeological materials, protein preservation pathways, and protein validation ^18^. Indeed, ancient proteins are complex mixtures of multiple protein groups, minerals, and other organic molecules like carbohydrates and lipids. These complex mixtures are also subject to a wide range of environmental factors over time, including the pH of a burial environment, groundwater, soil chemistry, and temperature. The resulting complex and heterogeneous PTMs may complicate the analysis of MS2 data. For example, Maillard reactions and advanced glycation end products may alter cleavage efficiency during enzymatic digestion ^48^. Glycation and lipidation are also labile PTMs, and they could be broken down during MS2 fragmentation, further affecting the assignment of PTM sites and PSMs ^49^.

Given these analytical challenges, one strategy is to leverage open searching provided by software such as Fragpipe ^50^. An open search is characterised by a wide precursor tolerance window, generally extending up to +500 Daltons, to account for mass shifts between experimental data and theoretical values. MSFragger manages the enlarged search space by using a fragment ion index, mass binning, and precursor mass ordering to optimise the retrieval and scoring during peptide-spectrum matching; then open searching outputs are annotated by PTM-Shepherd that utilises signal-to-noise ratio filtering, retention time shifts, MS2 spectra resemblance and the entirety of the Unimod modification database ^51^. Fragpipe also offers a workflow optimised for labile PTMs ^49^. While FDR controls are still challenging for open searching ^52^, it could be advantageous for the global discoveries of PTMs and damage patterns.

### Is palaeoproteomics data FAIR?

Lastly, like any other scientific datasets, palaeoproteomics data is progressing towards the FAIR principles ^53^. Archaeological data is generally findable, accessible, and reusable thanks to the establishment and adherence to data-sharing practices. Acquired raw files from mass spectrometers, processed peak lists, and search results are often readily available to download from public repositories such as PRIDE and MassIVE. This fosters transparency and collaboration within the research community. We would urge the community to continue prioritising data dissemination, as mass spectrometry pipelines are rapidly advancing. It is crucial to ensure reproducibility and create possibilities for future research to build upon current findings, considering archaeological materials are finite resources and often cannot be resampled.

However, achieving true interoperability can be challenging due to the inherent complexity and diversity of palaeoproteomics experiments. We would highlight the usefulness of routinely reporting bulk identification rates for complex, heterogeneous materials. This simple quality control matrix is integral for the comprehensive evaluation of search engines and the performance of identification pipelines. In addition, it is advantageous to report the number of assigned PSMs under a predefined FDR, total identified peptides, and the total assignments of protein groups. These figures could be included as supplementary information and uploaded to public repositories. They may also be useful in evaluating the effectiveness of database searching strategies, and determining whether it is worth exploring open and *de novo* sequencing methods.

Overall, we demonstrate that low identification rates are consistently observed in the 15 million ancient spectra from the 15 high-profile palaeoproteomics studies. These spectra without stories potentially result from search space management, the absence of appropriate databases, and suboptimal search parameters due to the degraded and modified nature of ancient proteins. While the findability, accessibility, and reusability of archaeological data appear to align with the FAIR principles, simple and standardised quality measures are still needed to navigate the complexity of palaeoproteomics data and enhance interoperability. While open searching and *de novo* sequencing are promising approaches to tackle these analytical challenges, they will require benchmarking, validation, and optimisation. Overall, these bioinformatics advances offer a pathway towards uncovering the hidden stories within the 94% of unidentified ancient spectra, and lessons learnt in palaeoproteomics may be valuable in other fields facing similar challenges with complex datasets.

## Ethics and consent

Ethical approval and consent were not required.

## Data Availability

All ancient datasets used in the paper are publicly available as follows: ProteomeXchange Consortium: palaeoproteomics data. Accession number PXD008647 ^19^. ProteomeXchange Consortium: palaeoproteomics data. Accession number PXD011008 ^20^. ProteomeXchange Consortium: palaeoproteomics data. Accession number PXD013838 ^22^. ProteomeXchange Consortium: palaeoproteomics data. Accession number PXD014342 ^23^. ProteomeXchange Consortium: palaeoproteomics data. Accession number PXD014657 ^21^. PRIDE: palaeoproteomics data. Accession number PXD014730 ^24^. ProteomeXchange Consortium: palaeoproteomics data. Accession number PXD018721 ^28^. ProteomeXchange Consortium: palaeoproteomics data. Accession number PXD020462 ^30^. ProteomeXchange Consortium: palaeoproteomics data. Accession number PXD021949 ^25^. ProteomeXchange Consortium: palaeoproteomics data. Accession number PXD022300 ^26^. ProteomeXchange Consortium: palaeoproteomics data. Accession number PXD022368 ^27^. ProteomeXchange Consortium: palaeoproteomics data. Accession number PXD024346 ^32^. ProteomeXchange Consortium: palaeoproteomics data. Accession number PXD024397 ^29^. ProteomeXchange Consortium: palaeoproteomics data. Accession number PXD024487 ^31^. ProteomeXchange Consortium: palaeoproteomics data. Accession number PXD024510 ^33^.
