# Supplementary material for: Spectra without stories: reporting 94% dark and unidentified ancient proteomes
Source: Open Res Eur. 2024 Apr 15;4:71. [Version 1] doi: 10.12688/openreseurope.17225.1 (PMC11187534; doi:10.12688/openreseurope.17225.1)
Supplement: Peer review history

# Reviewer report: Reviewer response for version 1

**DOI:** 10.21956/openreseurope.18615.r40925

**Published:** 2024-06-19

**Authors:** Virginia Harvey

**Affiliations:** 1 University of Chester, Chester, England, UK

Chiang et al. set about analysing almost 15 million ancient fragment ion (MS2) spectra from Nature and Science portfolios and looked for identification rates across the dataset. They found that over 94% of the queries remained unmatched/uncharacterised, resulting in only 6% of MS2 spectra that had actually been genuinely assigned to peptide sequence database matches. Of these, some still could have been contaminant matches, making the actual assigned MS2 spectra even less than this! This paper is very well written, clear, and makes the important case for effective data sharing practices to be employed by everyone in the field of palaeoproteomics (and beyond) to best allow for methodological developments, and to get the most output out of often irreplaceable ancient samples. I recommend indexing without changes.

Is the study design appropriate and does the work have academic merit?

Yes

Is the work clearly and accurately presented and does it cite the current literature?

Yes

If applicable, is the statistical analysis and its interpretation appropriate?

Not applicable

Are all the source data underlying the results available to ensure full reproducibility?

Yes

Are the conclusions drawn adequately supported by the results?

Yes

Are sufficient details of methods and analysis provided to allow replication by others?

Yes

Reviewer Expertise:

Palaeoproteomics, zoology, marine biology, ZooMS

I confirm that I have read this submission and believe that I have an appropriate level of expertise to confirm that it is of an acceptable scientific standard.

# Reviewer report: Reviewer response for version 1

**DOI:** 10.21956/openreseurope.18615.r40929

**Published:** 2024-06-12

**Authors:** Glendon Parker

**Affiliations:** 1 University of California, California, USA

**Spectra without Stories: reporting 94% dark and unidentified ancient proteomes.**

 The author’s conduct a meta-analysis of previously submitted paleoproteomic datasets that were published in high-quality / high-impact publications. These datasets for the most part originate from the network of highly productive collaborations centred around the Globe Institute at the University of Copenhagen. As such the datasets have consistent sample processing, data generation, and analytical workflows. The authors provide commentary about the degree of unidentified spectra in each sample and conclude that there are higher rates of unassigned spectra in paleoproteomics than more complex data dependent acquisition datasets.

 In terms of a meta-analysis the manuscript is not controversial, but also does not really address any of the hypotheses that could be tested given the advantage of the larger combined datasets available or suggested by the authors. An obvious and necessary item to correct is the lack of a confidence interval in the estimate of the size of the ‘dark paleoproteomes’. With no interval the number has no meaning.

 The following is an incomplete list of suggestions in no particular order.

1. The language used is tentative. The words ‘may’ or ‘could’ show up several times at critical moments and undercut the impact of the manuscript. The study is a meta-analysis, and so definitive statements may be difficult to justify. Ideally meta-analyses test hypotheses that can only be examined in a larger context. Nevertheless, the authors should be definitive when justified, but also refrain from speculation when appropriate.
2. The first paragraph of the introduction is overly detailed, and a broader framing of the author’s analysis is underdeveloped. We recommend the use of a background section to separate the necessary detail from the broader themes and hypotheses that are normally examined in an Introduction.
3. Is the use of high-quality PRIDE and MassIVE control datasets a relevant comparator for paleoproteomics? These are cell culture datasets. Fully differentiated tissues typically have lower yields and fewer detected proteins. Biological tissue proteomes provide less data but may be more relevant. Surely the authors have access to a modern skeletonized sample dataset.
4. The issue raised by the first reviewer is pertinent. The figure is attractive but not that informative. Likewise, data that would help provide context, such the age of the sample, or degradation indices such as deamidation, or median peptide length are left out. A relationship between PSM efficiency and total spectra number would be more informative and easily accessible. Likewise, a ratio of PSMs with and without PTM modifications would be informative as well.
5. The datasets, while representing a range of targets, are selective. Some such as pottery are under-represented. Other groups are conducting paleoproteomics from a broader range of sample types, processing strategies, instruments, and analytical workflows. They may not be as good in quality or impact, but conclusions that are drawn from a larger, broader dataset will be more representative, universal, and applicable.
6. The authors could provide more detail on what is meant by heterogeneity of PTMs. PTMs come in two distinct types, biological and exogenous chemical modifications. These have fundamentally different etiologies and impacts. It was gratifying to see Maillard reactions mentioned but these likewise should be expanded on.
7. Finally, it is probably best to refrain from mentioning a strategy for a refined data search strategy without doing it. It is a good idea and leaving it out will protect the authors from losing priority.

Sample degradation in paleoproteomic samples is a challenge at many levels, in addition to low yields, the chemistries at play increase variation in peptide targets, distributing the remaining peptide backbone over an increasing number of peptide species. Spectra discarded by PSM algorithms may be a rich source of additional data and older datasets may continue to provide information with new bioinformatic workflows, well after the samples have been processed and fragmentation data generated. We look forward to seeing this work unfold.

Is the study design appropriate and does the work have academic merit?

Partly

Is the work clearly and accurately presented and does it cite the current literature?

Partly

If applicable, is the statistical analysis and its interpretation appropriate?

No

Are all the source data underlying the results available to ensure full reproducibility?

Yes

Are the conclusions drawn adequately supported by the results?

Yes

Are sufficient details of methods and analysis provided to allow replication by others?

Yes

Reviewer Expertise:

Forensic Proteomics, Paleoproteomics, Biochemistry, Mass Spectrometry. Proteomic Genotyping, Proteomic Sex Estimation.

I confirm that I have read this submission and believe that I have an appropriate level of expertise to confirm that it is of an acceptable scientific standard, however I have significant reservations, as outlined above.

# Reviewer report: Reviewer response for version 1

**DOI:** 10.21956/openreseurope.18615.r39932

**Published:** 2024-05-10

**Authors:** Timothy Cleland

**Affiliations:** 1 Smithsonian Museum Conservation Institute, Suitland-Silver Hill, Maryland, USA

Chiang et al. evaluate the levels of spectral identification in paleoproteomic data that has been previously published in Science and Nature portfolio journals.

 "To address the question... Science and Nature Portfolio journals" I understand limiting the choices of journal, but it seems unhelpful to use predominantly publications that use MaxQuant for database searching. Mascot also tends to be rather conservative in its identifications and the majority of the field does not use it. Can you explore other publications that use alternative search strategies as well (e.g., Proteome Discoverer/Sequest, MSFragger, MetaMorpheus)?

 "The identification rates of palaeoproteomics datasets range from 0.47% to 12.61%" Can you give both the overall percentages and also split this by search algorithm because they aren't the same?

 Figure 1. Please use a colorblind accessible palette. These colors are difficult to distinguish and PXD014657 becomes almost invisible in multiple colorblind viewer types. Also, I think this figure would be better as a regular bar chart making the comparison easy without having to have an index. Alternatively add the ID rate % to the end of the bars instead. Also, add the first author of each publication with its PXD number for ease of comparison because the data availability section uses numerical citations without easy connection to the authors.

Is the study design appropriate and does the work have academic merit?

Yes

Is the work clearly and accurately presented and does it cite the current literature?

Yes

If applicable, is the statistical analysis and its interpretation appropriate?

Yes

Are all the source data underlying the results available to ensure full reproducibility?

Yes

Are the conclusions drawn adequately supported by the results?

Yes

Are sufficient details of methods and analysis provided to allow replication by others?

Yes

Reviewer Expertise:

Paleoproteomics, mass spectrometry, proteomics, bone

I confirm that I have read this submission and believe that I have an appropriate level of expertise to confirm that it is of an acceptable scientific standard.
